# Quantified planar collagen distribution in healthy and degenerative mitral valve: biomechanical and clinical implications

**DOI:** 10.1038/s41598-024-65598-w

**Published:** 2024-07-08

**Authors:** Mohammad Javad Sadeghinia, Robert Matongo Persson, Vegard Skalstad Ellensen, Rune Haaverstad, Gerhard A. Holzapfel, Bjørn Skallerud, Victorien Prot, Stig Urheim

**Affiliations:** 1 Department of Structural Engineering, Norwegian University of Science and Technology (NTNU), Richard Birkelands Vei 1A, 7034 Trondheim, Norway; 2 Department of Heart Disease, Haukeland University Hospital, Bergen, Norway; 3 Institute of Clinical Science, Medical Faculty, University of Bergen, Bergen, Norway; 4 Institute of Biomechanics, Graz University of Technology, Graz, Austria

**Keywords:** Degenerative mitral valve disease, Fibroelastic deficiency, Barlow’s disease, Collagen remodeling, Biomedical engineering, Tissues, Valvular disease, Tissue engineering

## Abstract

Degenerative mitral valve disease is a common valvular disease with two arguably distinct phenotypes: fibroelastic deficiency and Barlow’s disease. These phenotypes significantly alter the microstructures of the leaflets, particularly the collagen fibers, which are the main mechanical load carriers. The predominant method of investigation is histological sections. However, the sections are cut transmurally and provide a lateral view of the microstructure of the leaflet, while the mechanics and function are determined by the planar arrangement of the collagen fibers. This study, for the first time, quantitatively examined planar collagen distribution quantitatively in health and disease using second harmonic generation microscopy throughout the thickness of the mitral valve leaflets. Twenty diseased samples from eighteen patients and six control samples were included in this study. Healthy tissue had highly aligned collagen fibers. In fibroelastic deficiency they are less aligned and in Barlow’s disease they are completely dispersed. In both diseases, collagen fibers have two preferred orientations, which, in contrast to the almost constant one orientation in healthy tissues, also vary across the thickness. The results indicate altered in vivo mechanical stresses and strains on the mitral valve leaflets as a result of disease-related collagen remodeling, which in turn triggers further remodeling.

## Introduction

Degenerative mitral valve disease (DMVD) affects approximately 2% of the population and is characterized by mitral regurgitation (MR), defined as systolic retrograde flow from the left ventricle into the left atrium^1,2^. DMVD exhibits pronounced microstructure alterations in the affected mitral valve leaflets^3^. The disrupted microstructure is associated with mechanically incompetent leaflets, which leads to a dysfunctional mitral valve and MR^4,5^.

**Figure 1:**
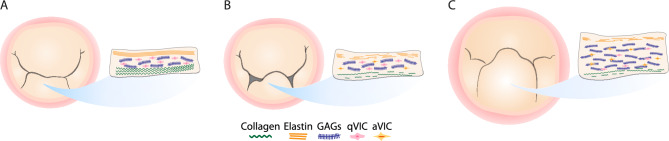
(**A**) Illustration of the mitral valve. Mitral valve leaflets are comprised of three main distinct layers; (from the ventricular side) a collagen-rich fibrosa, a spongiosa layer consisting of mostly glycosaminoglycans (GAGs), and an elastic atrialis. The entire leaflet is also populated with quiescent valvular interstitial cells (qVIC). (**B**) Mitral valve with fibroelastic deficiency (FED) and (**C**) Barlow’s disease. Both diseases are characterized by fragmented collagen and elastin fibers with activated valvular interstitial cells (aVIC), exhibiting myofibroblast-like behavior.

The mitral valve leaflet is a well-organized structure comprising of three distinct layers: the collagen-dense fibrosa, the spongiosa, rich in proteoglycans, and the elastic atrialis (Fig. 1A). The entire thickness is populated by valvular interstitial cells (VIC), a distinct type of cell, specific to cardiac valves^6^. DMVD has arguably two distinct phenotypes: fibroelastic deficiency (FED) and diffuse myxomatous degeneration, also known as Barlow’s disease (BD)^7–14^. Previous studies have found that FED typically presents with localized myxomatous degeneration in relatively thin leaflets, which leads to MR, as illustrated in Fig. 1B. FED is also associated with a deficiency of collagen and elastin fibers. BD is marked by an excess amount of tissue with diffused myxomatous degeneration, (Fig. 1C), and the collagen and elastin fibers are fragmented with an excessive accumulation of proteoglycans. Both phenotypes also exhibit activated VICs with myofibroblast-like behavior and smooth muscle $\alpha$-actin^8,10,12,15,16^.

Among the components of the extracellular matrix, collagen is the most important as it is the main mechanical contributor to the proper functioning of mitral valve leaflets (MVL)^17–19^. A disruption of the collagen network leads to significantly altered biomechanics and consequently malfunction of the mitral valve leaflet. Histological examination has traditionally been the standard method of microstructure analysis in DMVD^8,10,12,15,16^. The histological analyses examine the tissue laterally, due to the thin leaflets of the mitral valve, like the schematics in Fig. 1. While it provides valuable insights, it is not adequate for a full analysis of the biomechanics and the function of the mitral valve leaflets, as these are governed by the planar organization of the collagen fibers^20–26^. How the collagen fibers are dispersed and oriented in the plane orthogonal to the histological sections is of decisive importance, both in terms of biomechanics and clinical implications.

Various advanced imaging techniques have been employed to quantify the alignment and orientation of collagen fibers. Small-angle light or X-ray scattering (SALS/SAXS)^23,27,28^, polarized spatial frequency domain imaging (pSFDI)^24,25^, and second harmonic generation (SHG)^5,21,26^ imaging are the most notable ones. SAXS and pSFDI provide a large field of view and have fast image acquisition. However, these techniques examine an aggregated signal, which means all the collagen fibers over the entire thickness of the tissue are aggregated in one acquisition plane. On the other hand, SHG provides excellent lateral and transmural resolutions, enabling a thorough examination of collagen organization, particularly in case of pronounced collagen alteration as associated with the phenotypes of DMVD. Regardless of the method of investigation, previous studies on planar distribution of collagen fibers have primarily relied on healthy tissues or tissues from animals^26,29^.

In this study, we examine differences in the planar collagen alignment and preferred orientations between FED, BD and healthy tissues to demonstrate the marked alteration in collagen remodeling. To the best of our knowledge, this is the first comprehensive description of the planar collagen distribution in degenerative mitral valve disease. This is of particular interest because the planar collagen distribution dictates the mechanical behavior of the leaflets and thus the functions of the mitral valve. This study has the potential to be used for more optimal design of tissue-engineering heart valves. The results presented here can also inform an in silico analysis for more realistic modeling towards personalized medicine in heart valve surgery. The biomechanical impact of planar collagen distribution could help provide pathophysiological explanations for the observed differences between fibroelastic deficiency and Barlow’s disease. It has the potential to guide treatment strategies, aimed at both achieving optimal blood flow dynamics and restoring normal biomechanics of the mitral valve^14^.

## Results

### Collagen fiber distribution from SHG acquisition

**Figure 2:**
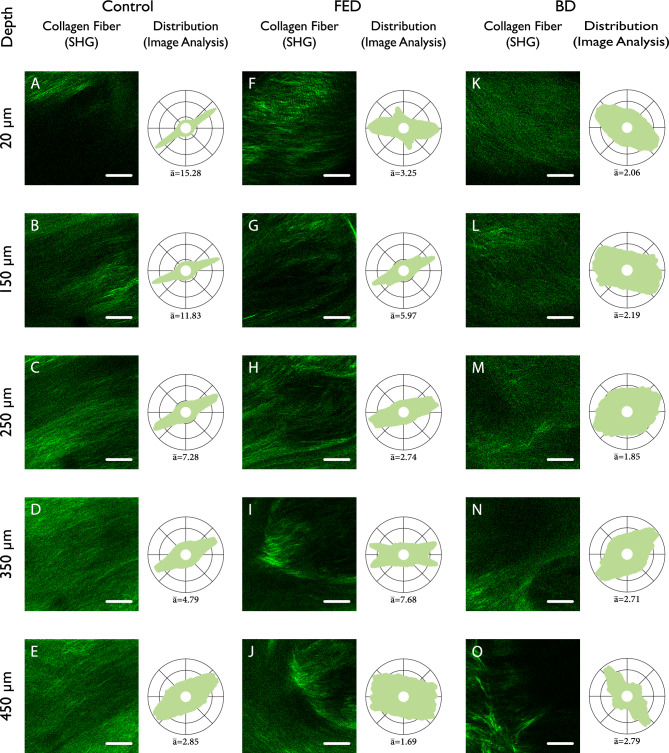
SHG acquisition of collagen fibers for a representative (**A**–**E**) control, (**F**–**J**) FED and (**K**–**O**) BD, all from the P2 segment, and their respective collagen fiber distributions. The images are acquired at different depths from the ventricular side; (**A**, **F**, **K**) 20μm, (**B**, **G**, **L**) 150μm, (**C**, **H**, **M**) $250$ μm, (**D**, **I**, **N**) $350$ μm, (**E**, **J**, **O**) $450$ μm. The scale bars are 100μm.

Figure 2 shows SHG images and the corresponding quantified distribution for three representative samples of control (healthy), FED and BD. The SHG images are acquired at different depth from the ventricular side of the leaflets. Each SHG image is quantified by an angular fiber distribution through image analysis. Figure 2 illustrates significant differences in collagen fibers between the FED and BD groups compared to the control group. These differences include changes in collagen fiber alignment and the preferred orientations, which are investigated quantitatively between all the samples in control, FED and BD group.

### Collagen fiber alignment

**Figure 3:**
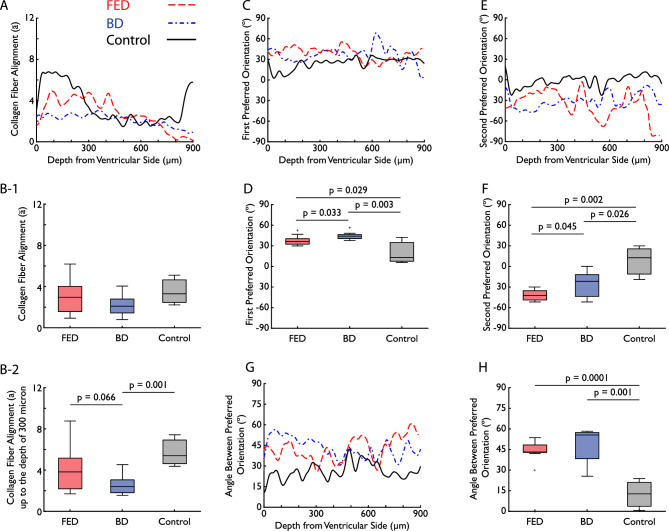
The variation across thickness and boxplots of (**A**, **B**-1 ,**B**-2) collagen fiber alignment, (**C**, **D**) first and (**E**, **F**) second preferred orientations, and (**G**, **H**) the angle between preferred orientations for control (black solid curve), FED (red dashed curve) and BD (blue dash-dotted curve). Plots A, C and E are the average of all samples within each group, and boxplots B, D and F are calculated averages for each patient. For collagen fiber alignment, B-1 is the boxplots from the average patient value for the entire acquisition depth, while B-2 only considers the first 300$\upmu$m, which corresponds to the fibrosa layer. In the boxplots, data outliers are represented by a ‘ + ’ sign.

The collagen fiber distributions are characterized by an average fiber alignment parameter $(\overline{a})$ that represents the degree of collagen fiber alignment. The higher parameter $\overline{a}$ means the higher alignment of the collagen fibers. As shown in Fig. 2, collagen fibers in FED and BD undergo marked changes compared to control group. Collagen fibers in the control sample (Fig. 2A–E) are highly aligned, while collagen fibers in FED (Fig. 2F–J) and BD (Fig. 2K–O) are more dispersed. The alignment of collagen fibers ($\overline{a}$) based on SHG analysis was calculated for each depth and averaged between samples within each group. The result is shown in Fig. 3A. In the control group, collagen fibers were highly aligned to a depth of $300\mu m$ from the ventricular side, which also corresponds to the collagen rich fibrosa layer. For FED samples, the collagen fibers were on average less aligned than in the control samples in the fibrosa layer, indicating a higher dispersion and remodeling of the collagen fibers. In both the control and the FED samples, the alignment of collagen fibers decreased to the same level after the depth of $300\mu m$. Interestingly, BD samples exhibit a nearly constant and low alignment of collagen fiber throughout the thickness on average, which is the same level as of control and FED samples after the depth of $300\mu m$. This indicates that the highly aligned collagen fibers up to the depth of $300\mu m$, observed in the control group, were more dispersed in BD samples.

To compare collagen fiber alignment between samples, an averaged fiber alignment parameter is calculated for each patient over the entire measured depth and compared using the ANOVA test and boxplots (See Fig. 3B-1). For the two samples from the same patient in FED and BD, the average value between samples was used in the ANOVA test. For the healthy tissue, the average value between segments A2 and P2 was used for each subject (see Fig. 7). The control, FED and BD differ from each other in the distribution of collagen fiber alignment, but no significant difference was observed. From Fig. 3, it can be seen that the control group has distinct layers; the $300\mu m$ thick fibrosa layer with highly aligned collagen fibers ($\overline{a}\approx 6$) and other layers with less alignment and higher dispersion ($\overline{a}\approx 2$). The FED showed less alignment in the fibrosa layer but was distinct from other layers. However, there was no difference in the orientation of collagen fibers across the thickness in BD. The detailed collagen fiber alignment throughout the depth is shown in Table 1. The average of collagen fiber alignment ($\overline{a}$) for control samples was $3.54\pm 0.79$ versus $2.8\pm 0.79$ and $2.15\pm 0.43$ in FED and BD, respectively. Moreover, the alignment of collagen fibers in healthy tissue is significantly higher up to a depth of $300\mu m$, corresponding to the fibrosa layer, as shown in Fig. 3A. As presented in Fig. 3B-2, the control group has significantly higher collagen fiber alignment compared to the BD.

**Table 1:** Parameters for the collagen fiber distribution for healthy, FED and BD samples based on depth from the ventricular side. Values are reported as mean (one standard deviation).

	Collagen fiber alignment, $\overline{a } (-)$			First preferred orientations $(^\circ )$			Second preferred orientations $(^\circ )$			Angle between preferred orientations $(^\circ )$		
	FED	BD	Control	FED	BD	Control	FED	BD	Control	FED	BD	Control
0–100μm	3.34 (1,78)	2.54 (0.46)	6.02 (1.37)	40 (5)	40 (4)	11 (11)	− 35 (7)	− 35 (7)	− 11 (11)	39 (5)	52 (4)	22 (9)
100–200μm	4.02 (0.56)	2.43 (0.28)	6.47 (0.97)	48 (6)	28 (2)	15 (4)	− 20 (4)	− 45 (3)	− 7 (7)	44 (4)	52 (3)	22 (6)
200–300μm	4.48 (0.63)	2.56 (0.19)	4.76 (0.53)	42 (8)	33 (3)	23 (5)	− 14 (4)	− 36 (2)	− 3 (3)	36 (4)	47 (4)	26 (6)
300–400μm	3.93 (0.54)	2.85 (0.76)	2.61 (0.49)	40 (8)	43 (3)	26 (4)	− 36 (5)	− 30 (5)	1 (6)	35 (4)	43 (3)	25 (7)
400–500μm	3.54 (1.51)	2.38 (0.28)	2.14 (0.61)	48 (8)	34 (6)	28 (7)	− 26 (8)	− 32 (5)	− 2 (4)	35 (5)	38 (3)	30 (4)
500–600μm	2.41 (0.37)	2.11 (0.35)	2.01 (0.46)	29 (5)	38 (10)	28 (12)	− 59 (15)	− 26 (4)	− 6 (11)	51 (5)	38 (4)	33 (4)
600–700μm	2.2 (0.29)	1.83 (0.69)	1.76 (0.69)	25 (4)	55 (11)	31 (6)	− 38 (7)	− 32 (8)	1 (7)	36 (6)	39 (3)	30 (5)
700–800μm	0.95 (0.83)	1.57 (0.54)	2.24 (0.51)	32 (14)	33 (11)	30 (2)	− 19 (17)	− 23 (3)	7 (4)	49 (7)	39 (5)	23 (4)
800–900μm	0.31 (0.62)	1.05 (0.27)	3.86 (1.46)	29 (0)	25 (11)	30 (5)	− 57 (0)	− 20 (10)	5 (7)	42 (0)	43 (7)	25 (8)
Average	2.8 (0.79)	2.15 (0.43)	3.54 (0.79)	37 (6)	36 (7)	25 (6)	− 34 (7)	− 31 (5)	− 2 (7)	41 (4)	43 (4)	26 (6)

**Figure 4:**
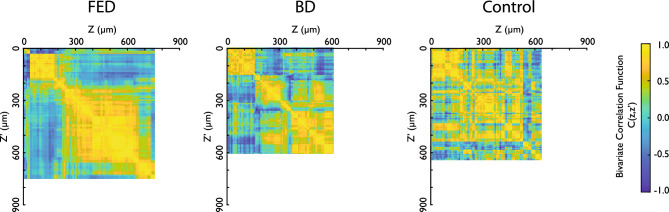
Bivariate correlation function C(Z, Z′) of collagen fiber alignment at different depths from the ventricular side, visualized as a color map for FED, BD and control group, where a value of 1 indicates a strong positive correlation and -1 a strong negative correlation. The blank area is due to missing information on one or more samples at that specific depth (see Fig. 5).

Furthermore, for each pair of depths $z$ and $z{\prime}$, the bivariate correlation function *C*(*Z*, *Z*′) for the collagen fiber alignment, $a(z)$ and $a\left({z}{\prime}\right)$, was calculated based on Eq. (3) and is presented in Fig. 4. The control group has a broad region of higher correlation spanning a significant portion of the depth. This consistently high correlation suggests a uniform collagen fiber alignment, which likely reflects the natural, undisturbed state of collagen fiber distribution in the valve leaflet. For the FED samples, the correlation map exhibits a clear concentration of higher correlation coefficients, represented as warmer colors, around the initial and middle depths. This creates two clusters with high correlation, which suggests a strong intra-layer correlation of the collagen fibers within the fibrosa and spongiosa, while there is a weak correlation between these two layers in contrast to the control group. A similar pattern is observed in the BD state; however, the correlation values are lower than in the FED samples and at some depths there is no correlation at all. Therefore, the intra-layer correlation is weaker in BD, suggesting more diffused degeneration.

### Preferred orientations of collagen fibers

As shown in Fig. 2, collagen fibers in the control group are oriented in a certain preferred orientation. There is only one preferred orientation of ${30}^{\circ }$ away from the circumferential direction, which is also almost constant throughout the depth. On the contrary, FED and BD samples exhibited a different collagen distribution. The collagen fiber distribution showed two distinct preferred orientations and, in some cases, e.g., Fig. 2M, it is difficult to identify a clear preferred orientation for collagen fibers. Moreover, the preferred orientations vary by depth within FED and BD groups.

The averaged variations of the first and second preferred orientations are presented in Fig. 3C and E, respectively, with the numerical value listed in Table 1. Similar to collagen fiber alignment, an averaged first and second preferred orientation is calculated over the entire measured depth for each patient, and compared using the ANOVA test and boxplots (see Fig. 3D and F). There is a significant difference between all groups in both the first and second preferred orientation. Interestingly, the two preferred orientations of the control group were close to each other, in contrast to FED and BD. Therefore, it seems reasonable to assume there is a single family of collagen fibers in the control group, while there are two distinct families of fibers in FED and BD.

To provide an unbiased test of this hypothesis, the difference between two preferred orientations at each depth was calculated for each sample and then averaged within each group, as shown in Fig. 3G. The boxplot in Fig. 3H is based on the averaged sample difference between two preferred orientations. The angle between preferred orientations for FED and BD was significantly different from control, while there was no significant difference between FED and BD. The median of the difference between two preferred orientations for FED and BD was ${43}^{\circ }$ and ${55}^{\circ }$, respectively, while this value for the control was ${13}^{\circ }$. It is evident that collagen fibers in FED and BD have a larger angle between the preferred orientations, making the two preferred orientations more pronounced compared to the control group.

### Distribution map of collagen fibers

**Figure 5:**
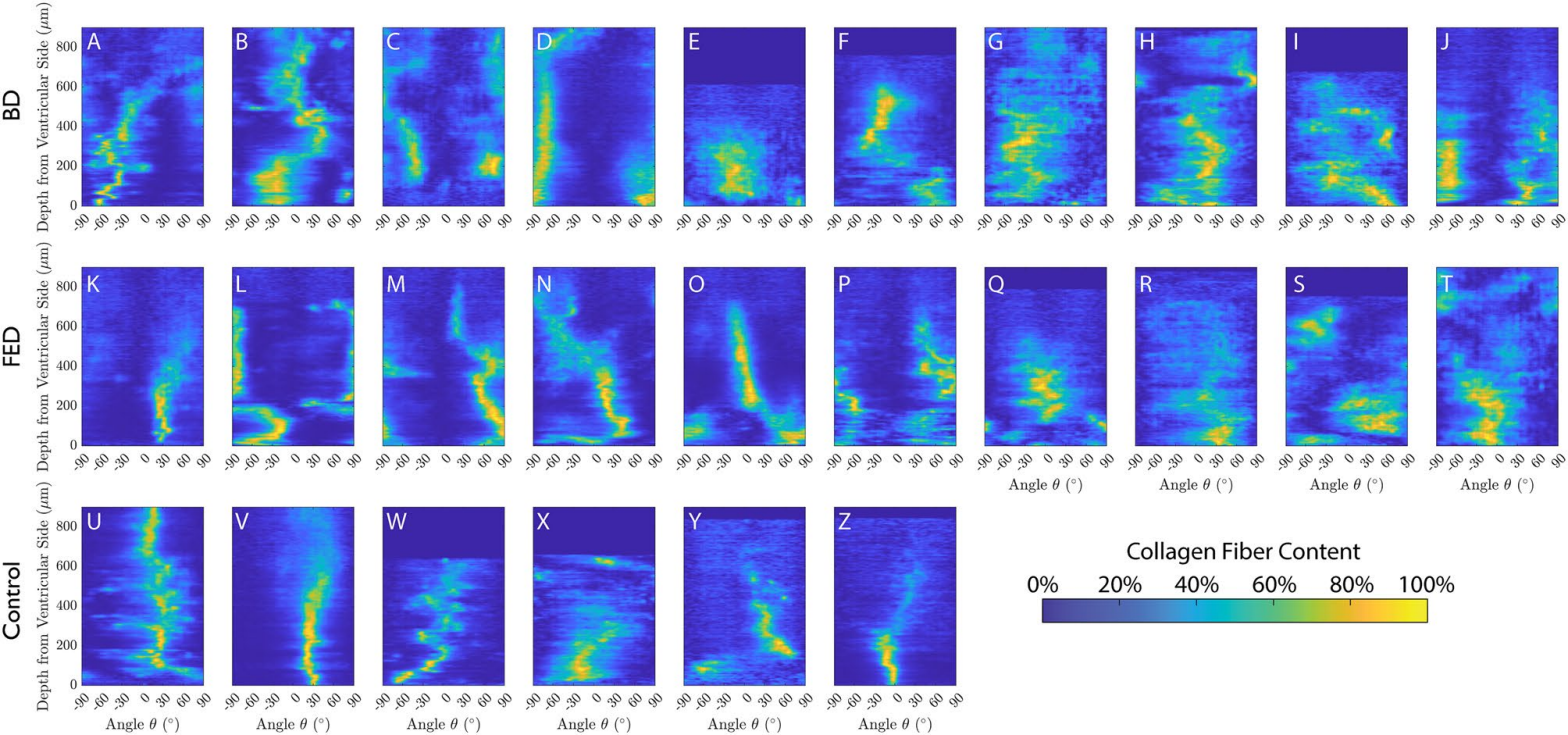
3D distribution map of collagen fiber content differentiated by angle (x-axis) and depth (y-axis) for (**A**–**J**) BD, (**K**–**T**) FED and (**U**–**Z**) the control group. F and G are from the same BD patient and Q and R are from the same FED patient. The order of the letters corresponds to the order of the patient IDs. For the control, U, W and Y are from the anterior leaflets of the first, second, and third subjects, respectively. V, X and Z, in turn, are from the posterior leaflets of the first, second, and third subjects, respectively.

A distribution map of collagen fiber content, differentiated by orientation and depth can be generated. The amount of collagen content can be represented by color. The results for all the investigated samples are presented in Fig. 5. The main preferred orientation, with the highest collagen content (indicated by the yellow color in Fig. 5), remains almost constant through the thickness for most of the control samples. However, for BD samples, it varies. For FED samples, some, e.g., Fig. 5K and O, exhibit an almost constant preferred orientation, similar to healthy tissues, while the rest display varying preferred orientations that is similar to BD samples. The same results are also evident from averaged plot in Fig. 3C and E. In conclusion, the healthy samples can be represented by one preferred orientation, while for BD and most of the FED samples, there are two preferred orientations.

### Comparison of SHG versus histological analysis

**Figure 6:**
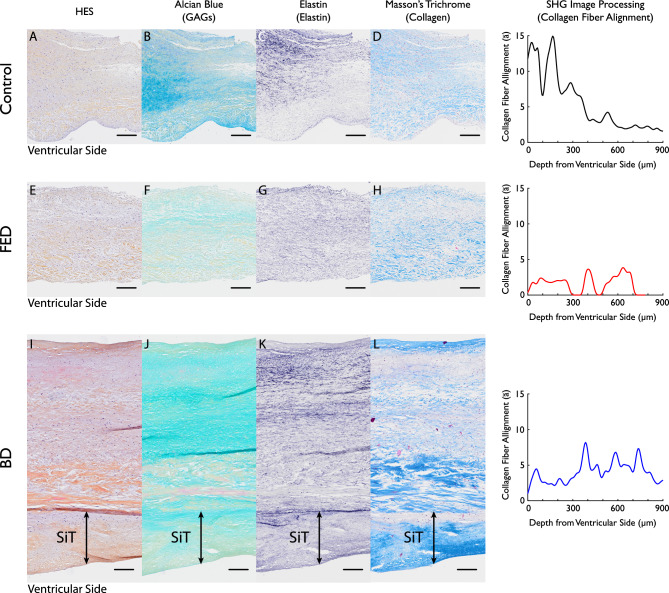
Histopathological analysis of a representative (**A**–**D**) control, (**E**–**H**) FED and (**I**–**L**) BD MVL samples and their respective collagen fiber alignment from SHG image processing. All the samples are from P2 segment. The histological sections are stained with (**A**, **E**, **I**) hematoxylin–eosin saffron (HES) for general examination, (**B**, **F**, **J**) Alcian Blue for glycosaminoglycans (GAGs), (**C**, **G**, **K**) Elastin for elastin fibers and (**D**, **H**, **L**) Masson Trichrome for collagen fibers. Superimposed tissue layer (SiT) is observed in the BD sample indicated with SiT, see (**I**–**L**). Note that histological sections are sectioned transmurally for a side-view, while SHG examines the planar arrangement of collagen fibers in the orthogonal plane with respect to the histological sections. SHG imaging acquisition is performed on the side where histological sections are dissected to allow comparison between SHG and histological analyses. The scale bar is 200μm.

In addition to SHG analyses, histological analyses were also performed for several samples. Figure 6 shows the histological analyses for three representative sample compared to their corresponding quantified collagen fiber alignment based on SHG. Histological analyses (Fig. 6G,H,K and L) show that collagen and elastin fibers and the layers are disrupted in FED and BD and that there is an accumulation of GAGs in BD (Fig. 6J). Recently, it has been demonstrated that in DMVD a fibrous layer, called superimposed tissue (SiT) layer, is formed on the original leaflet^16^ possibly induced by the abnormal mechanical stresses^30^. In this present study, a SiT layer was found in the BD sample on the ventricular side (Fig. 6I–L).

As shown in Fig. 6, collagen fibers in the control sample were highly aligned to a depth of 300$\mu m$ from the ventricular side. Based on the histology section, this region corresponds to the fibrosa layer, the collagen-rich layer of MVL (Fig. 6A–D). While collagen fibers are present in other layers, particularly in the atrialis (Fig. 6D), SHG analyses showed that these fibers are less aligned and more dispersed outside the fibrosa layer (Fig. 3A and Table 1). It is unlikely that this information can be derived from histological analyses.

For the representative FED sample, SHG analyses indicates a low collagen fiber alignment across the thickness of the leaflet. Several regions exhibit a random distribution without any preferred orientation, i.e., $$\overline{a}=0$$. Compared to control samples, FED samples lack regions with high alignment, even though collagen fibers are scattered up to $$650$$$$\mu m$$ depth (Fig. 6H). These findings are consistent with SHG analyses, showing dispersed collagen fibers to a depth of $$700$$$$\mu m$$.

For the BD sample, SHG analyses revealed low collagen fiber alignment at $0-300 \mu m$ depth followed by increased alignment of collagen at a depth of $300-800 \mu m$, although more dispersed compared to the control sample. The histological analyses indicate that these two regions correspond to the SiT and the fibrosa layer (Fig. 6I, J, L).

## Discussion

Collagen fibers are the main contributor to the structural integrity and mechanical competency of MVL^17–19^. In DMVD, MVL is associated with a disrupted microstructure of collagen fibers^8,12,15,16^. This is in turn associated with mechanically incompetent leaflets^4,5^. While the predominant traditional investigation method has been histopathological analysis, this technique primarily involves transmural sections and lateral examination. However, MVL is a planar structure with collagen fibers arranged in layers orthogonal to the histological sections. Therefore, histopathological analysis offers limited insight into how collagen fibers are distributed in each layer and how the orientation varies between these layers. This information is of particular interest for biomechanics and therefore the function of the MVL is determined by the alignment of collagen fibers in the planar organization^20–24,26,31^.

In this study, we used SHG as an artifact-free 3-dimensional imaging modality of collagen fibers in the main phenotypes of DMVD, FED and BD. We then used appropriate image analysis methods to quantify collagen organization at each layer up to $900\mu m$ depth from the ventricular side. We focused on the collagen fiber alignment and the preferred orientation of collagen fibers.

Previous studies have demonstrated increased synthesis and degradation of collagen fibers in myxomatous degeneration^32–36^. The present study shows that FED and BD have lower alignment in the fibrosa layer and two distinct preferred orientations. The lower alignment is possibly due to degradation and the secondary orientation can be attributed to the remodeling in an attempt to reinforce the tissue. The secondary direction also suggests that the mechanical stresses and strains on the leaflets are altered in the in vivo state of FED and BD, as growth and remodeling occur in the direction of the mechanical strain^37,38^.

The present study also provides valuable information on the mechanics of the diseased MVL. With today’s more conservative tissue resections, it is difficult to obtain sufficiently large samples for mechanical testing, which represents the gold standard for material modeling of biological tissues. However, previous studies on animal tissue and healthy mitral valves concluded that the tissue mechanics is determined by the distribution and strength of collagen fibers^20–24,26,31^. In our previous study on the mechanical behavior of MVL from one FED and one BD patient, we found considerably weaker collagen fibers, compared to other studies on healthy MVL^5^, which is to be expected from histological analyses due to collagen fragmentation^8,10,12,15,16^. This study now provides a detailed collagen fiber distribution of eighteen patients. The weaker and more dispersed collagen fibers in FED and BD result in higher mechanical strains in all directions, particularly circumferentially, where collagen fibers of healthy tissue are aligned (Fig. 2A–E, Fig. 3C and E). Higher strain leads to the activation of valvular interstitial cells, which in turn leads to more remodeling^37–40^.

Moreover, the present study showed for the first time that there may be a secondary family of collagen fibers in diseased MVL. Understandably, this has not been reported before as histological analyses of microstructure in DMVD offer only a lateral view, which is limited in terms of biomechanical analysis. This finding is particularly important in the novel in silico studies investigating mitral regurgitation,^41–43^, as a quantified analysis of the microstructure in DMVD is a necessity. In addition, the current study will enable a more accurate mechanical modeling of the MVL^5^. Combining the findings of the current study with in silico analysis, may provide further insights into how the quantified remodeling leads to mitral valve prolapse and potentially guide future treatment. Understanding the complex relationship between biomechanics, perturbed collagen structure and disease states could have significant implications for providing long-term repair strategies^6^. While current surgical interventions for primary MR have demonstrated success in restoring valve competency and enhancing blood flow dynamics, it remains unclear whether this will reverse microstructure remodeling of the mitral valve leaflet^6,14^. A more comprehensive understanding of the remodeling processes in FED and BD could facilitate customized repairs, aiming not only to achieve optimal blood flow dynamics but also to restore the normal biomechanics of the mitral valve.

There is an ongoing debate as to whether BD and FED should be treated as two separate diseases or as variations within the same spectrum of disease. In this study, we used advanced imaging and image analysis to assess the remodeling of collagen fibers in MVL as the most important mechanical factor. The results showed similar remodeling of FED and BD, but with less alignment in BD. The increased remodeling observed in BD with the resulting deterioration in mechanical properties may be a factor contributing to the complexity of the disease and the higher reoperation rate observed compared to FED^44,45^. Furthermore, BD is characterized by diffuse myxomatous degeneration, while FED is associated with localized myxomatous degeneration. Studies have demonstrated that myxomatous leaflets are less stiff and more extensible, correlating with the low alignment observed in this research^4,5^. BD tends to develop earlier in life than FED, which is considered age-related^14^. The chronicity of the disease could potentially explain the difference in the extent of collagen alignment. Although different gene expression patterns were identified in the mitral valve leaflets of FED and BD, a significant overlap in gene expression was found, possibly indicating compensatory changes^46^. However, further genetic studies and immunohistochemical studies of the molecular and cellular events are needed^3^. For example, GAGs play a major role in the increased thickness of BD samples (Fig. 6J) and may contribute to layer delamination and other mechanical incompetency^6,47–49^. Nevertheless, the results of the current study suggest that FED and BD, regardless of whether they are the same or different diseases, both result in similar collagen remodeling. Because collagen is the main load carrier, remodeling will result in further alteration of the mechanical leaflet strains, and therefore most likely cause further remodeling, setting a vicious cycle. Nevertheless, the causal relationship between DMVD remodeling and the occurrence of valvular regurgitation remains unclear.

One limitation of the current study may be the number of samples but should be seen in relation to the time- and resource-intensive process of sample preparation, image acquisition and processing. It is worth noting that the investigation of each sample consists of more than 150 images throughout the thickness. Therefore, more than 1500 images from FED and 1500 images from BD were investigated in this study. Another limitation is that the BD samples are significantly thicker than those from the control and FED groups. The imaging depth of $900\mu m$ only covers the SiT and the fibrosa in the BD sample (Fig. 6I–L) or fibrosa and part of the spongiosa in the absence of SiT, while it covers the entire tissue thickness in the control and FED samples. To mitigate this, in addition to variation across thickness (Fig. 3A,C,E and G), we also investigated the pool of quantified variables using the boxplots (Fig. 3B,D,F and H). The collagen-rich fibrosa layer, which is the main load bearer, has been included in all the groups. Another limitation is that one must be careful when interpreting the absolute angle values (Fig. 3C and E). Even if the orientation of the samples and the circumferential direction were recorded at the time of explantation and the sample for SHG was mounted with special care for the orientation, the absolute angle value is prone to error. For this reason, we also investigated the angular difference between the first and second preferred orientation at each depth for each sample as an unbiased methodology (Fig. 3G). After all, MVL is not a passive spectator. The transition zone from the annulus to the leaflets expresses smooth muscle $\alpha$-actin in healthy tissue^50^, and the valvular interstitial cells transform into an activated myofibroblast‐like phenotype, expressing smooth muscle‐associated contractile proteins^12,51,52^. While the study focused on collagen fibers, as the main component of the passive behavior of the MVL, further research is needed to investigate how DMVD affects the active behavior of the MVL and mitral apparatus.

In conclusion, this study is the first to examine the planar collagen remodeling of MVL in DMVD. In contrast to cross-sectional histological analyses, planar examination is important to correlate the microstructural disruptions in DMVD with the mechanical deficiency of MVL leading to MR. BD is associated with a complete dispersion of collagen fibers in the fibrosa layers, while FED is less dispersed, yet more than in healthy tissue. In both FED and BD there was evidence of what needs to be characterized as a secondary family of collagen fibers. We believe that this study provides a deeper understanding of the interplay between DMVD, microstructural disruption and the biomechanics of MVL, which is central to both pathophysiologic understanding and the development of future treatment strategies.

## Methods

### Sample acquisition

The study population consisted of eighteen patients undergoing mitral valve surgery for severe MR, of which nine were diagnosed with FED (age $62\pm 11$ yrs, two females and seven males) and nine with BD (age $57\pm 11$ yrs, five females and four males). Samples from three deceased individuals (age $52\pm 12$, two females and a male) acted as controls. BD was defined by echocardiographic examinations and intraoperative analysis as leaflet thickening with diffuse myxomatous degeneration, annular dilatation and billowing of several leaflet segments, whereas FED was characterized by leaflet prolapse due to chordal rupture in the absence of myxomatous degeneration.

**Figure 7:**
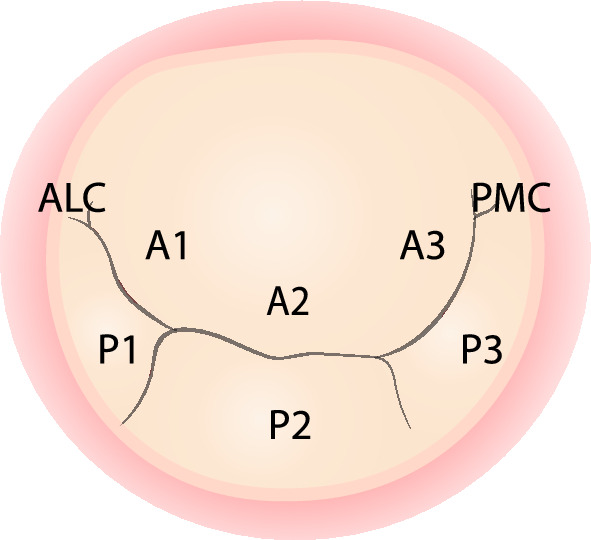
Illustration of the mitral valve showing the three scallops of the posterior leaflet (P1–P3) and the corresponding segments of the anterior leaflet (A1–A3). ALC refers to the anterolateral commissure and PMC refers to the posteromedial commissure.

Mitral valve tissue was obtained from valvular excisions during elective mitral valve surgery, by repair (n = 17) or replacement (n = 1). The mitral valve repair procedures included annuloplasty, valvuloplasty with resection, and neo-chordae implantation. Ten samples were acquired from each group of FED and BD patients (two samples were excised from the same patient once in each group), amounting to a total of twenty DMVD samples. Upon explantation, the samples were marked according to their orientation, with the circumferential direction defined as parallel to the annulus and the radial direction as orthogonal to the circumferential direction. The samples were then snap frozen and stored in the Bergen Cardiovascular Biobank (ID: 2014/828). For analysis, samples were transported in a liquid nitrogen dry shipper (Worthington CX100, US) and stored in the dry shipper until thawed for analysis. Control mitral valves were obtained post-mortem from patients undergoing autopsy and with no known prior cardiac disease. A total of six samples were obtained from three individuals, one from the P2 and one from the A2 segment from each patient (Fig. 7). After excision, the control mitral valves were transported to the testing facility using the same protocol as for surgically excised tissue.

The study was approved by the Regional Committee for Medical and Health Research Ethics (Project ID: 2016/1132 and 2016/2073). All examinations were performed according to the rules for the investigation of human subjects set out in the Declaration of Helsinki. All study participants provided written informed consent.

### Sample preparation

Before analysis, the samples were thawed at room temperature and submerged in 1 $\times$ PBS. The samples were then fixed in a 4% formaldehyde solution for 18 h. After tissue fixation, several samples were dissected into two sections: a radial section for histopathological analysis and another for SHG imaging of collagen structure. The rest were only used for SHG imaging of collagen fibers due to their smaller size.

The specimens for histology underwent a continued fixation process, followed by graded ethanol dehydration and paraffin embedding. A 5 *m* thick section was then cut from the specimen and stained with Hematoxylin, Eosin and Saffron (HES), Elastin for elastin, Alcian Blue for glycosaminoglycans (GAGs) and Masson Trichrome for collagen.

After tissue fixation, the SHG samples were chemically cleared with either 1:2 Benzyl Alcohol:Benzyl Benzoate (BABB) solution^53^ or SeeDB clearing^54^. SHG imaging is usually limited to in-depth imaging up to $200\mu m$ from the surface, however imaging depth can be increased substantially by utilizing tissue clearing^55^. The tissue clearing renders the tissue translucent and allows in-depth tissue imaging using SHG, with minimal morphological alteration.

In BABB clearing, the samples are dehydrated based on graded absolute ethanol; 50%, twice 70%, twice 95% and twice 100%, each step lasts 30 min. Then, the sample is immersed in 1:1 absolute ethanol:BABB solution for 4 h and finally in BABB solution for 18 h. In the SeeDB clearing process, the sample is first incubated at 25 °C in solutions of 20%, 40%, and 60% (weight:volume) fructose in 0.1 × PBS. It is then immersed for 12 h in an 80% concentration solution followed by another 12 h incubation in 100% (weight:volume) fructose in distilled water. Finally, the sample is incubated with fully saturated fructose solutions in distilled water for 24 h. During the incubation process, the solution is rotated at 4 rpm using a tube rotator (PTR-35 Grant Instruments, UK).

### Histology and second harmonic generation microscopy

**Figure 8:**
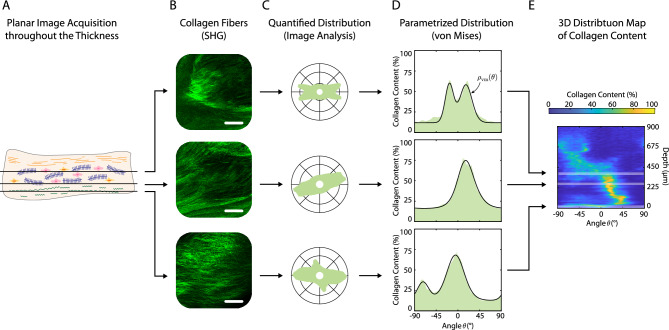
(**A**) Acquisition of planar images of collagen fibers using second harmonic generation microscopy (SHG) throughout the thickness from the ventricular side, with a z-step of 5μm. (**B**) Corresponding SHG images showing collagen fibers at different depths. The scale bars are 100μm. (**C**) Quantitative analysis showing the distribution of collagen fibers, collagen content at a specific angle for each acquisition depth. (**D**) Applying the circular von Mises distribution to the collagen fiber distribution to parameterize it. (**E**) Illustration of collagen content with color and layering of various corresponding distributions to create a 3D distribution map of collagen fiber content differentiated by angle (x-axis) and depth (y-axis).

The histological sections were scanned with the Olympus VS120 slide scanner (Olympus, Japan) at 20 $\times$ magnification. SHG imaging was performed using Lecia SP8 (Leica Biosystems, Germany) with Leica HCX IRAPO 25 $\times$ objective and numerical aperture of 0.95. The laser excitation was set at 890 nm to excite the collagen fibers. Collagen imaging was performed from the ventricular side of MVL up to a depth of 900$\mu m$ toward the atrial side through the thickness, with z-steps of 5$\mu m$, as illustrated in Fig. 8A. To compensate for the scattering, the laser power was increased linearly. For the samples with histological sectioning, SHG imaging was performed near the side from which the histological section was dissected. The image field of view was 465$\mu m\times$ 465$\mu m$ with a resolution of 1024 $\times$ 1024 pixels (Fig. 8B).

### Quantification and statistical analysis

We use image analysis to determine the degree of collagen fiber alignment and preferred orientations through the thickness. To determine the distribution of collagen fibers in each image (Fig. 8C), a 2D Tukey window is applied to the image, which is then Fourier transformed and multiplied by its conjugate complex to find the power spectrum density. This allows the fiber direction to be distinguished based on frequency and orientation. Then, a wedge-shaped filter with a range of − 89°–90° and an increment of 1° is used to extract the fiber orientations at a certain angle θ. Finally, to smooth the data, a moving average filter with a range of 7° is applied to the angle θ. In this way, we quantify the collagen fiber distribution for each image at a specific depth, as shown in Fig. 8C. The distribution is then fitted by two families of fiber using a von Mises distribution (Fig. 8D) defined as

1 $${\rho }_{\text{vm}}\left(\theta \right)=w\frac{1}{\pi }\frac{\mathit{exp}\left\{{a}_{1}\mathit{cos}\left[2\left(\theta -{\alpha }_{1}\right)\right]\right\}}{{I}_{0}({a}_{1}) }+\left(1-w\right)\frac{1}{\pi }\frac{\mathit{exp}\left\{{a}_{2}\mathit{cos}\left[2\left(\theta -{\alpha }_{2}\right)\right]\right\}}{{I}_{0}\left({a}_{2}\right)}.$$

Here ${\rho }_{\text{vm}}\left(\theta \right)$ is the von Mises distribution characterized by $\alpha$ and $a,$ the mean fiber angle and fiber alignment parameter, respectively, and the subscript denotes the first or second fiber family, while $w$ is the weighting factor with values from $0$ to $1$ and ${I}_{0}$ is the zero-order modified Bessel function of the first kind.

We can now define an average fiber alignment parameter that represents the degree of collagen fiber alignment at each depth, i.e.

2 $$\overline{a }={a}_{1}w+{a}_{2}\left(1-w\right).$$

The higher the value of $\overline{a}$, the higher the alignment and the lower the dispersion. We also define the isotropic distribution, similar to a previous study^56^ in which the alignment of collagen fibers was set to zero and no preferred orientation was assigned.

Each SHG acquisition at a specific depth has a collagen density plot (Fig. 8D). This value can also be displayed in color and then the distribution of all images can be stacked to create a 3D distribution map showing the content of collagen fibers at a specific depth and in a specific orientation, as shown in Fig. 8E.

### Statistical analysis

To compare the distribution of planar collagen fibers in control, FED and BD, the quantified averaged degree of alignment ($\overline{a}$) and the preferred orientations (${\alpha }_{1}$, ${\alpha }_{2}$) were determined for each sample at every depth. The parameters for each specific depth were then averaged across samples within each group. Additionally, the difference between the two preferred orientations at each depth was calculated for each sample and subsequently averaged within each group. This approach ensures an unbiased examination, as the absolute value of the preferred orientation differs from one specimen to another.

Moreover, an average value over the entire depth is calculated for each patient. For the patients with two samples, the average value of the two samples was used. Then, the averaged values were compared between groups using the ANOVA test. In the entire statistical analysis of the fiber orientations, we leave out all isotropic layers, i.e., $a=0$ and therefore no preferred orientation. We use circular statistical analysis because the orientation data are circular in nature and all computations are performed using custom scripts in MatLab and circStat package in MatLab^57,58^.

To quantify the correlation of collagen fiber alignment at different depths of the mitral valve leaflets, we utilized the bivariate correlation function *C*(*Z*, *Z*′). For each pair of depths $z$ and *Z*′ , the bivariate correlation function is calculated as

3 $$C\left(z,{z}{\prime}\right)=\frac{{\sum }_{i=1}^{n}\left(X\left({z}_{i}\right)-\overline{X}\left(z\right)\right)\left(X\left({{z}{\prime}}_{i}\right)-\overline{X}\left({z}{\prime}\right)\right)}{\sqrt{{\sum }_{i=1}^{n}{\left(X\left({z}_{i}\right)-\overline{X}\left(z\right)\right)}^{2} {\sum }_{i=1}^{n}{\left(X\left({{z}{\prime}}_{i}\right)-\overline{X}\left({z}{\prime}\right)\right)}^{2}} }.$$

Here, $X({z}_{i})$ and $X\left({{z}{\prime}}_{i}\right)$ represent values of measurements of collagen fiber alignment at depths $z$ and $z{\prime}$ for the $i$-th sample, $\overline{X}\left(z\right)$ and $\overline{X}\left(z{\prime}\right)$ are the mean values of these measurements at the respective depths and $n$ is the total number of samples. The resulting function $C(z,z{\prime})$ provides a matrix in which each element quantifies the degree of correlation between collagen alignments at two specific depths. The values of this function range from − 1 to + 1, indicating perfect negative correlation on one side, perfect positive correlation at the other, and zero indicating no correlation, allowing detailed spatial analysis of correlation patterns within the tissue.

## Data Availability

The data that supports the results within this paper are available from the corresponding author upon request.
